# Successful treatment of immune‐related cystitis with bladder hydrodistension

**DOI:** 10.1002/iju5.12588

**Published:** 2023-04-03

**Authors:** Tsutomu Anraku, Hideki Hashidate, Tomoyuki Imai, Yoshiaki Kawakami

**Affiliations:** ^1^ Department of Urology Niigata City General Hospital Niigata Japan; ^2^ Department of Pathology Niigata City General Hospital Niigata Japan; ^3^ Present address: Department of Urology, Molecular Oncology Niigata University Asahimachi‐dori 1‐757, Chuo‐ku Niigata City Niigata 951‐8510 Japan

**Keywords:** hydrodistension, immune checkpoint inhibitor, immune‐related cystitis, interstitial cystitis, steroid

## Abstract

**Introduction:**

Although immune checkpoint inhibitors offer significant therapeutic benefits to patients with advanced cancer, they can also cause a variety of immune‐related adverse events. As immune checkpoint inhibitors are being widely used, rare immune‐related adverse events are being reported.

**Case presentation:**

A 70‐year‐old man with advanced salivary duct carcinoma was treated with pembrolizumab following radiotherapy. After receiving two doses of pembrolizumab, the patient experienced symptoms such as micturition pain and hematuria. Immune‐related cystitis was suspected, and the patient underwent a bladder biopsy and bladder hydrodistension. Histological analysis revealed non‐neoplastic bladder mucosa with CD8‐positive lymphocyte‐dominant inflammatory cell infiltration, consistent with immune‐related cystitis. The patient's bladder symptoms improved postoperatively without steroid administration.

**Conclusion:**

Although steroids are commonly administered to treat immune‐related adverse events, bladder hydrodistension may be a promising treatment option for immune‐related cystitis to avoid administration of steroids, which may impair the therapeutic effect of immune checkpoint inhibitors.

**Keynote message**

Immune‐related cystitis is a rare irAE. It is often successfully treated with steroids, but bladder hydrodistension may also be helpful. It may be worth trying bladder hydrodistension for immune‐related cystitis to avoid the side effects of steroids.

**Abbreviations & Acronyms**
BPS: bladder pain syndrome
CR: complete response
CT: computed tomography
HPF: high power field
IC: interstitial cystitis
ICI: immune checkpoint inhibitor
irAE: immune‐related adverse event
MP: methylprednisolone
N/A: not available
PD: progressive disease
PR: partial response
PSL: prednisolone
SD: stable disease
TIA‐1: T‐cell intracellular antigen 1

## Introduction

ICIs have revolutionized cancer treatment and have become an integral part of the standard of care for many cancers. However, ICIs often trigger side effects due to autoimmune mechanisms, which are referred to as irAEs. IrAEs can affect many organs throughout the body, and the probability of developing irAEs ranged from 54% to 76% in a meta‐analysis.^1^ Meanwhile, irAEs rarely affect the bladder, and only 12 cases of immune‐related cystitis have been reported so far.^2^, ^3^, ^4^, ^5^, ^6^, ^7^, ^8^, ^9^, ^10^, ^11^, ^12^ Steroids are recommended for the treatment of irAEs according to the guidelines,^13^ and most cases of immune‐related cystitis are also treated with steroids. However, whether steroids impair the therapeutic effect of ICIs remains controversial.^14^ Previously, a case of immune‐related cystitis in which bladder symptoms improved after bladder biopsy was reported.^5^ In that report, the authors noted that unintentional bladder hydrodistension during the biopsy might have contributed to the improvement in bladder symptoms. Herein, we describe a case of immune‐related cystitis that was successfully treated with bladder hydrodistension.

## Case presentation

**Fig. 1:**
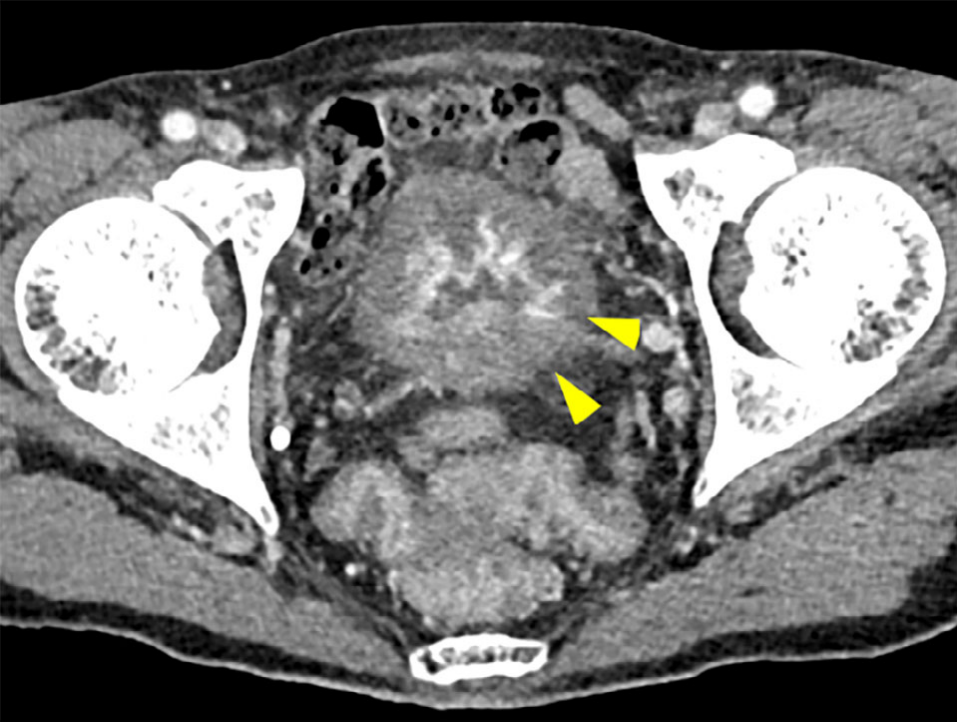
Pelvic imaging with contrast‐enhanced CT. The bladder wall is thickened with contrast enhancement of the mucosa (arrowhead).

**Fig. 2:**
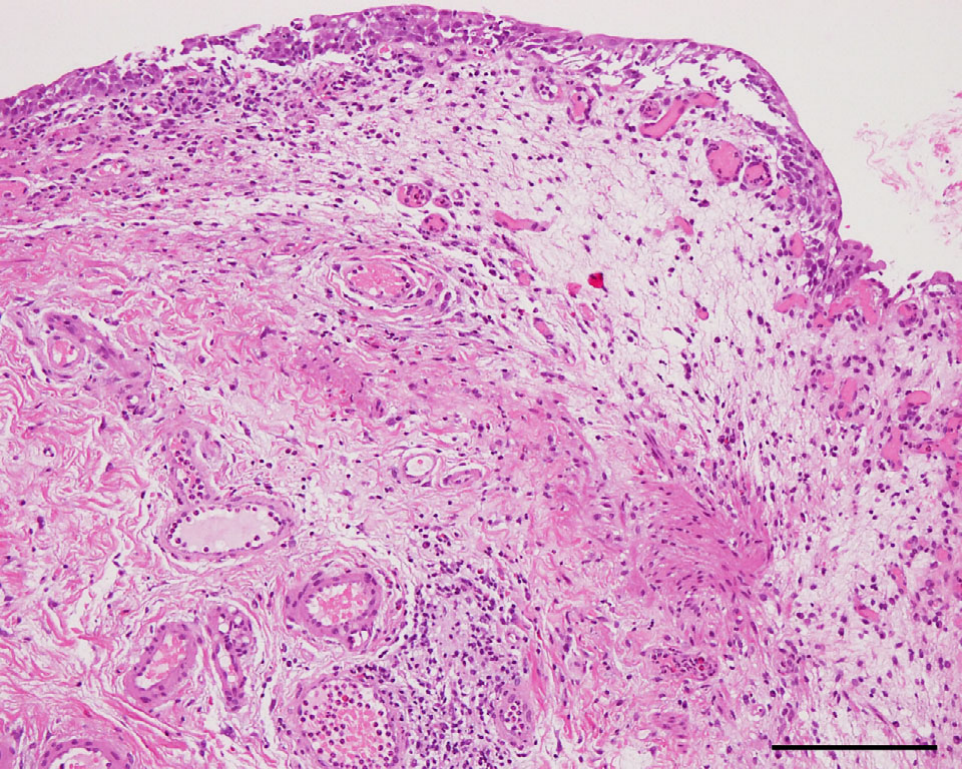
Hematoxylin and eosin staining of the bladder tissue. Non‐neoplastic bladder mucosa with epithelial detachment, interstitial edema, vascular hyperplasia, and inflammatory cell infiltration is observed in a hematoxylin–eosin‐stained specimen of the bladder. Scale bar indicates 200 μm.

**Fig. 3:**
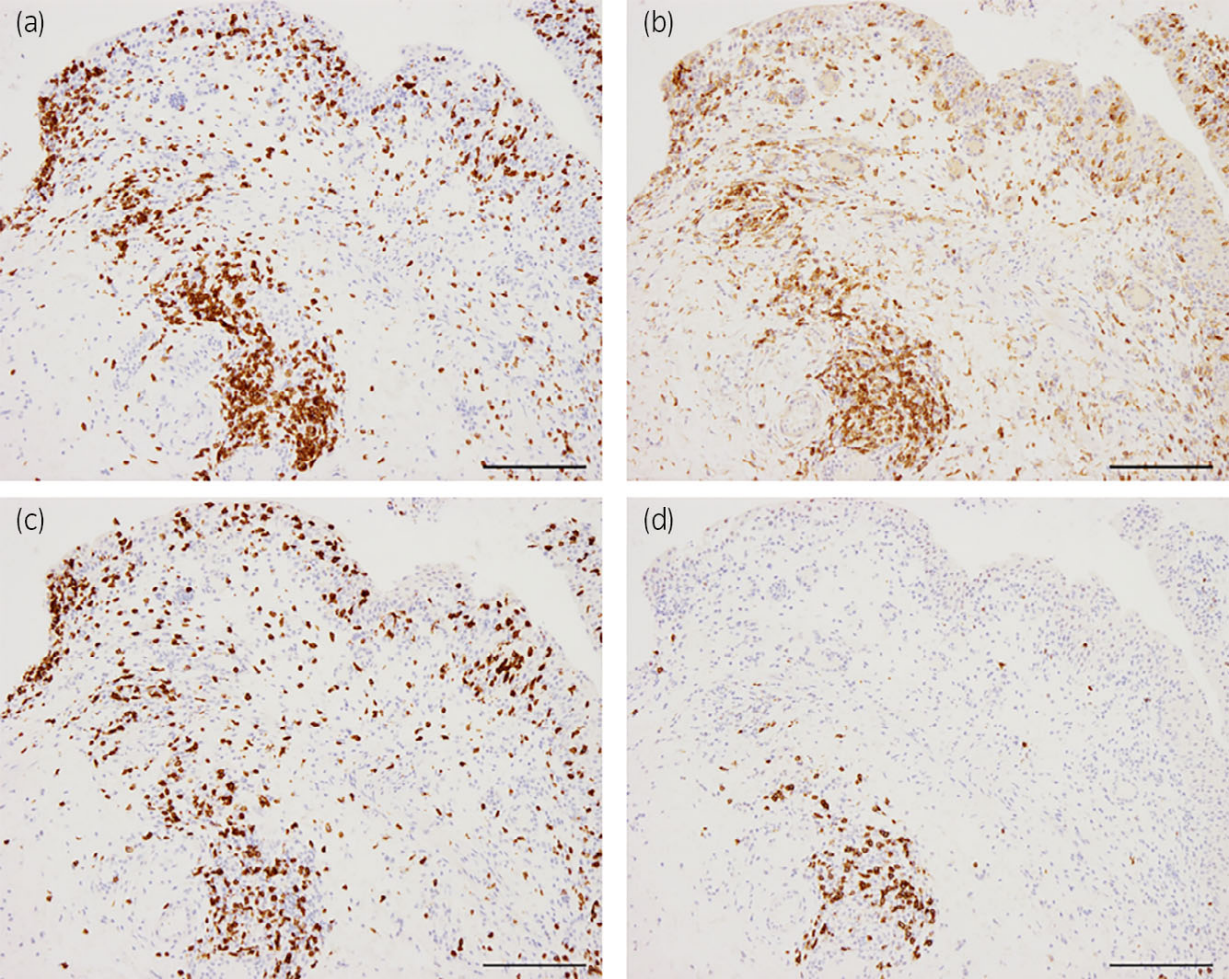
Immunohistochemical results in the bladder tissue. (a) CD3; (b) CD4; (c) CD8; (d) CD20. Scale bar indicates 200 μm.

A 70‐year‐old man with a history of hypertension, but not on oral medication, was diagnosed with salivary duct carcinoma (T4bN0M0, stage IVB). The patient received 70 Gy of radiotherapy, followed by immunotherapy with pembrolizumab at a dose of 200 mg every 3 weeks. Two weeks after the second administration of pembrolizumab, the patient developed fatigue, micturition pain, urinary frequency, and gross hematuria. CT revealed a thickened bladder wall and strongly enhanced bladder mucosa (Fig. 1), and cystoscopy revealed diffuse mucosal edema and bladder redness. Urinalysis revealed a red blood cell count of >100/HPF and a white blood cell count of 20–29/HPF. No causative bacteria were detected in the urine cultures, and the urine cytology was negative. Despite the discontinuation of pembrolizumab and administration of antibiotics and anticholinergic drugs, the urinary tract pain worsened to grade 3 based on the Common Terminology Criteria for Adverse Events v5.0. He urinated more than 30 times a day and his functional bladder capacity was less than 50 milliliters. Bladder biopsy and bladder hydrodistension under spinal anesthesia were performed approximately 2 months after the onset of the symptoms. Before the biopsy, the bladder was distended three times with saline at a pressure of 80 cm H_2_O for 3 min. The maximum bladder capacities during the distensions were 120, 140, and 140 mL. Mucosal bleeding in the posterior wall of the bladder was observed after the first distension (Video S1). Histological analysis showed non‐neoplastic bladder mucosa with epithelial detachment, interstitial edema, vascular hyperplasia, and inflammatory cell infiltration (Fig. 2). Immunohistochemical analysis revealed the infiltrated inflammatory cells to be positive for CD3 and CD8, partially positive for CD4 and CD20, indicating that cytotoxic T cells were predominant (Fig. 3). Based on the clinical course, we diagnosed immune‐related cystitis and considered the administration of steroids. However, the patient's symptoms were alleviated when the urethral catheter was removed on the second day after the endoscopic surgery. CT performed a month after surgery showed mild shrinkage of the parotid tumor. The patient underwent a repeat needle biopsy of the tumor, and no viable cancer cells were observed in the histopathological analysis. He has been monitored carefully, and there has been no recurrence of cancer or bladder symptoms 11 months after surgery.

## Discussion

The applications of ICIs have expanded to various cancers. As the use of ICI increases, there will be more opportunities to experience various irAEs. Therefore, familiarity with irAE management is crucial for clinical oncologists. Common irAEs include endocrine, gastrointestinal, lung, skin, and musculoskeletal disorders. It has been reported that the frequency or breakdown of irAEs varies according to the ICI class, cancer type, and race.^15^, ^16^ Immune‐related cystitis is a rare irAE, which has been reported in only 13 cases so far, including the current case (Table 1). Most of them have been reported in Asia,^2^, ^3^, ^4^, ^5^, ^6^, ^7^, ^8^, ^10^, ^11^, ^12^ suggesting that immune‐related cystitis may be more common among Asians.

**Table 1:** Clinical information on the reported cases of immune‐related cystitis

Authors	Age	Sex	Country	Symptoms	Primary cancer	ICIs	ICI cycle	Best response	Treatment for immune‐related cystitis	ICI discontinuation	ICI rechallenge	Pathological findings
Ozaki *et al*.^2^	62	M	Japan	Urinary frequency, micturition pain, macroscopic hematuria	Lung cancer	Nivolumab	3	PD	MP 500 mg × 3 days, followed by PSL 0.5 mg/kg and tapered	Yes	Nivolumab with PSL	Epithelial detachment, interstitial edema, slight lymphocytic infiltration
Shimatani *et al*.^3^	50	M	Japan	Urinary frequency, micturition pain	Lung cancer	Nivolumab	7	PD	PSL 1 mg/kg/day	Yes	Nivolumab	N/A
Shimatani *et al*.^3^	60	M	Japan	Urinary frequency, dysuria	Lung cancer	Nivolumab	12	SD	ICI discontinuation	Yes	Nivolumab	N/A
Ueki *et al*.^4^	78	F	Japan	Urinary frequency, micturition pain	Lung cancer	Pembrolizumab	6	PR	PSL 0.5 mg/kg/day	Yes	No	Infiltration of CD8^+^ and/or TIA‐1+ lymphocytes, PD‐L1+ urothelium
Yajima *et al*.^5^	47	M	Japan	Urinary frequency, micturition pain	Lung cancer	Nivolumab	18	PR	Bladder biopsy	No	N/A	Eosinophil‐ and plasma cell‐dominant infiltration
Tu *et al*.^6^	53	M	China	Urinary frequency, micturition pain, macroscopic hematuria	Lung cancer	Sintilimab	3	PR	MP 1 mg/kg/day	Yes	No	Lymphocyte‐dominant inflammation, interstitial tissue hyperplasia
Zhu *et al*.^7^	51	M	China	Urinary frequency, urgency, dysuria	Lung cancer	Nivolumab	5	CR	MP 160 mg/day	Yes	No	Infiltration of CD3^+^ and CD8^+^ lymphocytes
Zhu *et al*.^8^	48	M	China	Urinary tract irritation	Intrahepatic cholangiocarcinoma	Nivolumab	3	PD	Steroid hormones	Yes	Atezolizumab	Chronic inflammation, mucosal erosion, proliferation of granulation tissues and fibroblasts
Schneider *et al*.^9^	61	F	France	Urinary frequency, bladder pain, urgency, diarrhea	Melanoma	(i) Pembrolizumab (ii) Ipilimumab and nivolumab (iii) Nivolumab	(i) 2 (ii) 2 (iii) 4	PR	PSL 0.5 mg/kg/day	Yes	No	Regular epithelial layer with lymphocyte infiltration in intraepithelial and subepithelial connective tissue with lymphoid follicle
Wang *et al*.^10^	56	M	China	Urinary frequency, micturition pain, urinary incontinence	Gastric cancer	Sintilimab	5	CR	Chai‐Ling‐Tang (Sairei‐To)	No	N/A	Numerous infiltration of CD3^+^ and CD8^+^ cells into the urothelium, PD‐L1+ urothelium and subepithelial inflammatory cells
Obayashi *et al*.^11^	67	F	Japan	Urinary frequency, urinary tract pain	Breast cancer	Atezolizumab	4	PR	PSL 60 mg/body	Yes	N/A	Infiltration of CD8^+^ and/or TIA‐1+ lymphocytes, PD‐L1+ urothelium
Fukunaga *et al*.^12^	60	M	Japan	Penile pain, micturition pain	Lung cancer	Nivolumab	77	CR	MP 1 mg/kg/day Infliximab 5 mg/kg	Yes	No	N/A
Anraku *et al*. [present case]	70	M	Japan	Urinary frequency, micturition pain, macroscopic hematuria	Salivary duct carcinoma	Pembrolizumab	2	SD	Hydrodistension	Yes	No	CD3^+^ and CD8^+^ lymphocyte‐dominant infiltration, epithelial detachment, interstitial edema, vascular hyperplasia

In the current study, histopathology of the bladder showed CD8‐positive lymphocyte‐dominant infiltration, similar to previously reported cases of immune‐related cystitis.^4^, ^7^ This finding suggests that some antigens in the bladder are attacked by the immune system, which is consistent with immune‐related cystitis. In another case, an allergic mechanism was considered because eosinophil and plasma cell‐dominant infiltration was observed in the bladder histopathology.^5^ Although the precise mechanism of immune‐related cystitis remains unknown, steroids are most commonly administered as an effective treatment, similar to that for other irAEs. However, it may be better if irAEs can be treated without steroids, mainly for two reasons. First, there is concern that steroids may impair the anti‐tumor activity of ICIs owing to their immunosuppressive effects.^17^ Second, steroids induce side effects, such as hyperglycemia, hypertension, gastroduodenal ulcers, and infection. Based on the case report by Yajima *et al*., in which the potential effectiveness of bladder hydrodistension for immune‐related cystitis was mentioned, we successfully treated the current case with bladder hydrodistension and avoided steroid administration.

Although the precise mechanism of bladder hydrodistension remains unclear, one possible cause is that ischemia due to increased bladder wall tension leads to the degeneration of sensory nerve fibers.^18^ Bladder hydrodistension is usually performed to treat IC/BPS. IC/BPS presents with chronic bladder symptoms in the absence of confusable diseases.^19^ It is divided into Hunner‐type IC and BPS, the former characterized by Hunner lesions and inflammation of the bladder mucosa and the latter having neither of these features. Although no Hunner lesion was observed in cystoscopy in the current case, the clinical course and histological findings were similar to those of Hunner‐type IC, which is associated with autoimmune mechanisms in some cases. Furthermore, many animal models of autoimmune cystitis have been reported to mimic Hunner‐type IC.^20^ The efficacy of bladder hydrodistension usually lasts for only a few months.^19^ Careful follow‐up is required in the current case.

In conclusion, we present a case of immune‐related cystitis successfully treated with bladder hydrodistension. Although steroids are effective in the treatment of irAEs, bladder hydrodistension may be a promising treatment option for immune‐related cystitis. Further studies are required to elucidate the pathophysiology and management of this disease.

## Author contributions

Tsutomu Anraku: Conceptualization; writing – original draft. Hideki Hashidate: Investigation; supervision; validation. Tomoyuki Imai: Supervision. Yoshiaki Kawakami: Supervision.

## Conflict of interest

The authors declare no conflict of interest.

## Approval of the research protocol by an Institutional Reviewer Board

Not applicable.

## Informed consent

Written informed consent was obtained from the patient for the publication of this case report.

## Registry and the Registration No. of the study/trial

Not applicable.

## Supporting information


**Video S1.** Video of the bladder hydrodistension. The bladder was distended three times at a pressure of 80 cm H_2_O for 3 min. Mucosal bleeding in the posterior wall of the bladder was observed after the first distension.
